# Cytomegalovirus infection associated with livedo reticularis, erythromelalgia, and superficial venous thrombosis

**DOI:** 10.1016/j.jdcr.2024.05.003

**Published:** 2024-05-14

**Authors:** Hasina Maredia, Jenny Link

**Affiliations:** Department of Dermatology, Mayo Clinic, Rochester, Minnesota

**Keywords:** anticardiolipin, antiphospholipid, cytomegalovirus, erythromelalgia, infectious diseases, livedo reticularis

## Introduction

Livedo reticularis (LR) presents with violaceous net-like patches on the skin. It occurs most commonly over the lower extremities.^1^, ^2^, ^3^ LR is often a physiologic response secondary to cold temperatures causing vasospasm of the superficial cutaneous vasculature. However, LR can also be associated with medications, autoimmune conditions (lupus erythematosus or polyarteritis nodosum), or injury to the vascular lumen from circulating proteins such as cryoglobulins.^1^ Although less well-recognized, infections can also be associated with acute-onset LR.^2^^,^^3^ Antibodies to antigens from infections are hypothesized to induce intraluminal damage and thereby lead to LR.^2^ Early recognition of infectious etiologies of LR can lead to timely diagnosis and avoidance of unnecessary workup. In this case report, we describe cytomegalovirus (CMV) infection associated with LR as well as erythromelalgia and superficial venous thrombosis (SVT).

## Case report

**Fig 1:**
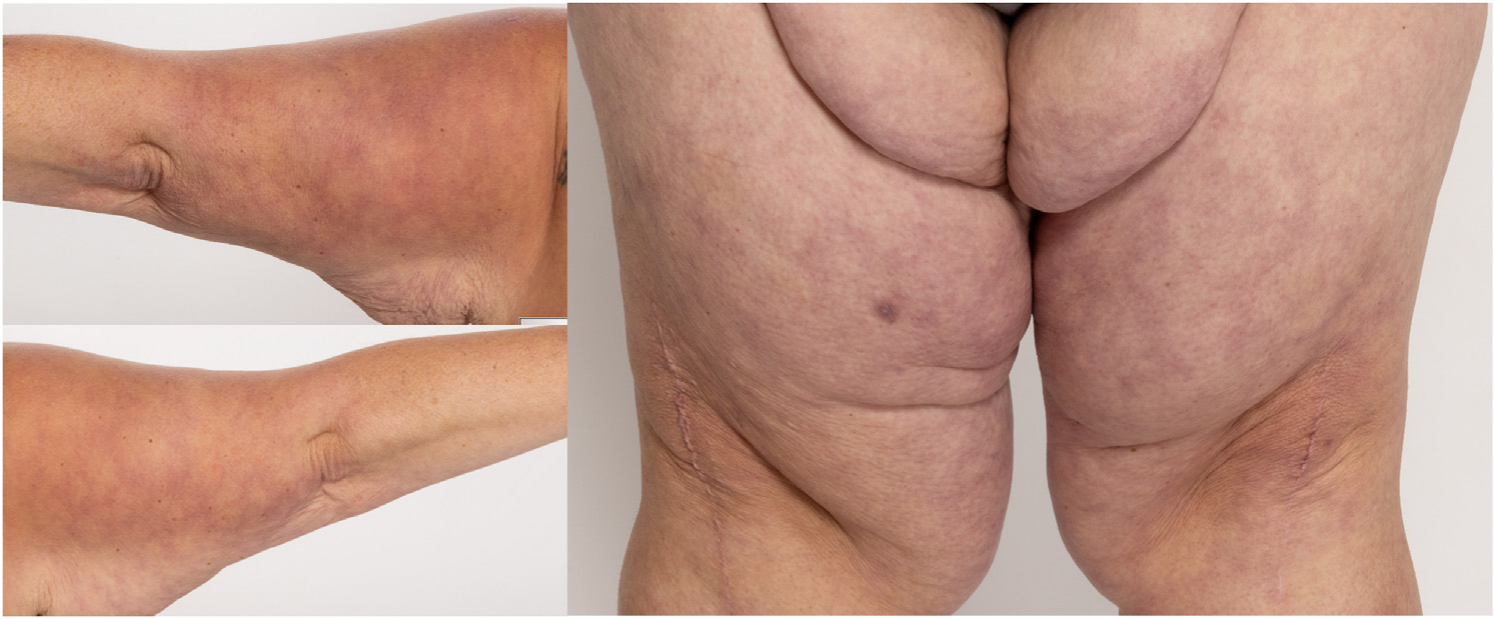
Over the posterior aspect of the upper arms and anterior aspect of the thighs bilaterally, there are violaceous, net-like patches consistent with livedo reticularis.

A 53-year-old-woman with no relevant past medical history presented to dermatology clinic with a 2-week history of a rash on the upper portion of the arms and thighs. The rash was asymptomatic and did not resolve with rewarming. Several weeks before rash onset, she had developed ongoing fatigue, fevers, headache, dyspnea, nausea, abdominal pain, and joint pain. During this period, she also experienced new episodes of the hands and feet becoming warm, red, and painful for a few hours before returning to normal. In the week before rash onset, she was diagnosed with a SVT on the lateral aspect of the right thigh and was started on apixaban. She worked at a daycare where she had sick contacts. She had no past medical history of thrombotic events, heavy menstruation, nor pregnancy complications. On examination, she had lacy, red-purple reticular patches with no nodules or ulcerations on the bilateral posterior aspect of the upper arms and anterior aspect of the thighs (Fig 1). Her skin findings were consistent with LR. She was also diagnosed with erythromelalgia, and amitriptyline-ketamine cream was recommended.

She was also evaluated in internal medicine, rheumatology, and infectious diseases. She had elevated CMV IgM and IgG. Her CMV viral load increased from 16,000 to 63,000 IU/mL within 9 days, associated with progression of her rash. She was found to have positive antinuclear antibodies of 1:320, but had negative extractable nuclear antigen panel and anti-double-stranded DNA antibodies. She had borderline positive IgM anticardiolipin antibodies (21 MPL, weakly positive: 15-39) but negative IgG anticardiolipin and lupus anticoagulant antibodies. Her anti-beta-2 glycoprotein I antibodies, antineutrophilic cytoplasmic antibodies, cryoglobulin, and complement levels were normal.

She was diagnosed with CMV. Her CMV viral load decreased over several weeks without treatment, and her systemic symptoms also started to improve.

**Fig 2:**
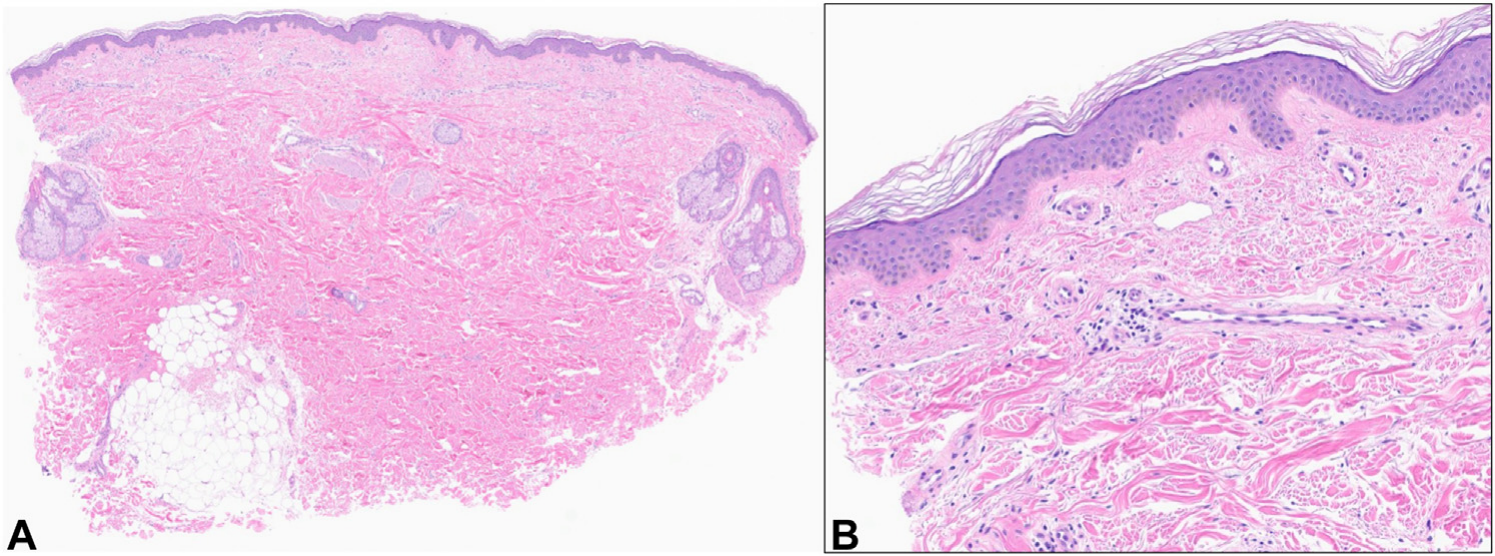
Over the posterior aspect of the left upper arm, skin punch biopsy stained for hematoxylin and eosin showed nonspecific sparse, superficial perivascular inflammation. (Digital magnification: **A,** 1×; **B,** 10×)

At 2-month follow-up, she reported persistent, diffuse joint pain and LR, but her other systemic symptoms had resolved. She had relief from erythromelalgia with amitriptyline-ketamine cream. Due to persistence of LR in the setting of joint pain, skin biopsies were performed of the patches on the upper portion of the left arm and left medial thigh. Skin biopsies demonstrated sparse superficial perivascular inflammation, consistent with LR (Fig 2). Serologic studies were performed. She had newly positive lupus anticoagulant antibodies, positive IgM anticardiolipin antibodies that had increased to 60.9 MPL (positive: 40.0-79.9), and weakly positive IgG anticardiolipin antibodies to 17.5 GPL (normal, <15). Her anti-beta-2 glycoprotein I antibodies, rheumatoid factor, anticyclic citrullinated peptide antibodies, anti-double-stranded DNA antibodies, extractable nuclear antigen panel, and complement levels were negative. She was found to have a heterozygous prothrombin G20210A mutation. Conservative treatments were recommended for her joint pain and LR, and she was continued on apixaban.

At 3-month follow-up, she had improvement in the joint pain and LR, and her repeat anticardiolipin and lupus anticoagulant antibodies were undetectable.

## Discussion

CMV has been associated with rare reports of vascular disorders, including LR.^2^, ^3^, ^4^ There have also been reports of CMV-associated cutaneous necrotizing vasculitis and acute hemorrhagic edema.^2^^,^^3^ In our case, we noted development of multiple vascular sequelae associated with CMV infection including LR, erythromelalgia, and SVT.

Although the exact mechanism of CMV-associated vascular injury has not been established, proposed mechanisms include damage to endothelial cells directly, induction of coagulation factors, and/or immune-mediated injury through induction of antiphospholipid antibodies.^2^ In this case, induction of antiphospholipid antibodies likely played a role in the development of her new-onset vascular conditions. During her CMV course, we noted borderline elevated IgM that significantly increased, with subsequent development of IgG antibodies and lupus anticoagulant antibodies. After her systemic symptoms resolved, these antibody levels returned to normal. Lupus anticoagulant levels can be falsely elevated when patients are on apixaban, but in this case, anticardiolipin IgM and IgG were also positive.^5^ The presence of heterozygous prothrombin G20210A mutation suggests a higher propensity to developing vascular injury and coagulopathy secondary to CMV.^6^

In summary, acute-onset LR in the presence of systemic symptoms should prompt consideration of infectious causes, including CMV. Early recognition of an infectious etiology can enable targeted history and lab investigation. In certain predisposed patients, CMV may be associated with additional vascular sequelae, including erythromelalgia and SVT. Although CMV usually self-resolves in immunocompetent patients, our case highlights that LR and reactive arthritis can persist after clearance of CMV with gradual improvement over time. Knowledge of the association of CMV with LR, erythromelalgia, and SVT is important for the dermatologist to bear in mind.

## Conflicts of interest

None disclosed.
